# Annotations

**Published:** 1894-09-08

**Authors:** 


### Annotations.

### "The Royal and Ancient Game" at St. Andrews.

Mr. A. J. Balfour has made the "Royal and ancient game of golf" famous throughout the civilised world. The *Times* added its benediction the other day, in an amusing leader; and now nothing is wanted but the general diffusion of knowledge of the game, and universal acceptance on the part of all outdoor mankind. The golf cultus constitutes the real religion of St. Andrews. The Duke of Roxburgh, we believe, is the present grand llama of the cult, and Mr. A. J. Balfour the high priest of the "Royal and Ancient Club." The club, which is lodged in a very handsome club-house at the eastern extremity of the "Links," has not less than a thousand members, and is very exclusive, very expensive, and very rich. At St. Andrews everybody plays golf, from the grandsire of ninety to the infant just out of his perambulator. The devotion of golf worshippers to their cult has probably never been equalled in the history of games. The Scottish squire, whose face has never been unduly elongated about the depression in agriculture, looks profoundly serious on the putting green; the retired colonel or old Indian civilian handles his driver and watches the construction of his "Tee" as if the fate of empires hung on his judgment. The most frivolous of flirts ceases to be frivolous on the "ladies' course," and addresses herself earnestly to the mysteries involved in the use of the "wooden putter." Even the clergyman, whose face is always grave from the gravity of his profession, manifests a gravity almost more than Sabbatic as he wields his driver for the last hole. At morning, at noon, and in the dewy twilight the game is unceasingly played. So large is the number of players that a solemn ballot is taken in the morning and again at midday, and the players take their places in the order of their ballotting luck. Such loyalty to a game is phenomenal, and demands the investigation of the philosopher, but more especially of the physiologist, whose business it is to take careful note of every circumstance which lays an enchaining hand from the cradle to the grave upon any considerable number of his fellow men.

### Unlicensed Asylums.

A few days ago a lady named Bale, supposed to be insane, was found thinly clad, in drenching rain, on a road near Bath. She was boarded in the house of an out-of-work clergyman named Shipley, who at some past time had held a living in Leicestershire, but now eked out his income by keeping weak-minded boarders. He had no licence for an asylum, and Miss Bale complained of the food, sleeping accommodation, and general treatment she received. Mr. Shipley protested that she was treated as a member of the family; but one would like to know how the family was treated before accepting this statement as satisfactory. There are too many "homes" like this in the country, and too many people ready to board their weak-minded and infirm relatives there, instead of sending them to proper institutions. The preference is due partly to family pride—they do not like to own to having a relative in an asylum; partly to a sincere belief that in a small household they will be treated with more consideration and personal regard than in a place where they are under the charge of trained, and therefore terrible, attendants. No greater blunder could be made. The licensed asylum is under supervision, it has for its head someone who has made a specialty of the various forms of lunacy, and the attendants maintain their position only if they show tact and consideration as well as firmness. In dealing with lunatics, as in dealing with children, the inexperienced are often cruel. They know neither when to humour nor when to control. And in such places as the Rev. Mr. Shipley's there is often more than inexperience to complain of. If a clergyman does not practise his profession, the reason is too often to be found in some moral weakness, which of itself condemns him as unfit for the charge of others, especially those of weak or morbid intellect. Unscrupulous cruelty is often the rule in such places where the complaints of the inmates will, on account of their insanity, be received with incredulity by their friends, and these friends, through a comprehensible but selfish and unpardonable cowardice, make their visits to the "home" as few and short as possible. Real homes there may be among such places, but there is no security against neglect and cruelty, and to put mental patients under even kind and well-meaning but untrained care, is to risk the aggravation of the disease where under proper treatment there might be alleviation or cure.

### The Pauper's Pipe.

The guardians of St. Saviour's, Southwark, have resolved to allow an ounce of tobacco to the infirm male inmates weekly, and either a quarter pound of tea or the same quantity of sweets to the female inmates in all the workhouses under their control. We welcome this concession. It will not be too costly a treat, and will do much to make the workhouse less intolerable to those within its walls. The workhouse should not be a desirable abode, but neither should it be unendurable to people who, however foolish and improvident they may have been, are not criminal, and are too old and broken-spirited to learn any lessons from the severest discipline. We would, for our own part, desire to see the "workhouse test" more regularly and sharply applied, in the interests both of the ratepayers and the paupers themselves, whose "outdoor relief" goes, as often as not, into the pockets of those they live with, and profits them hardly at all. But it is admitted that the little indulgences which the outdoor pauper can obtain compensate him for many discomforts and privations, and this concession of the occasional smoke will induce those who ought, for the sake of their own welfare, to be in the workhouse, to go there; while concurrent stringency in the granting of outdoor relief will induce the relatives of those who, by a fine distinction of pride, will not enter "the house," but will take all they can outside it from the parish, to make more strenuous efforts to support them. The two things ought to go together—decent provision, not generous, but not unreasonably hard, within the workhouse, and as little relief as possible outside. But might not something be done for the able-bodied who, by stress of circumstance, find themselves within the workhouse walls? Would it not be possible to give them some employment in the place—as attendants on officials, helping in repairs, working in the garden, and thus let them earn a little tobacco or tea? We do not think it would be altogether bad economy to let them have even some small money payment credited to them, so that when they leave they need not go into the world penniless. This is a matter on which no absolute rule can be laid down. The plan might be helpful, or it might be abused. But given a governor neither too soft-hearted nor too much a mere machine, and a Board of Guardians in earnest about solving the problem of pauperism, much might be done to make the pauper something more than the mere "burden on the rates which he is at present.

